# Transforming Diagnostic Precision in Periodontology Through Lab‐on‐a‐Chip Technology: A Scoping Review

**DOI:** 10.1155/ijod/8276749

**Published:** 2026-11-10

**Authors:** Midhu Manoj, Josh Jaison, Jothi Varghese

**Affiliations:** ^1^ Department of Periodontology Manipal College of Dental Sciences Manipal Academy of Higher Education Manipal India manipal.edu; ^2^ Data Analyst Department of Analysis New Energies Division Rystad Energy India Pvt. Ltd. Bengaluru Karnataka India

**Keywords:** biosensors, lab-on-a-chip, microfluidics, peri-implantitis, periodontitis, point-of-care testing, scoping review

## Abstract

**Background:**

Conventional periodontal diagnostics measures the damage that has already occurred rather than disease that is currently active. This gap has driven interest in point‐of‐care tools that read the mouth in real time. Lab‐on‐a‐chip (LOC) and microfluidic platforms are leading candidates for such chairside periodontal and peri‐implant testing, yet the supporting evidence has not been mapped in a structured way.

**Methods:**

A literature review was conducted in accordance with the Joanna Briggs Institute (JBI) methodology and reported following the Preferred Reporting Items for Systematic Reviews and Meta‐Analyses Extension for Scoping Reviews (PRISMA‐ScR). Electronic databases inclusive of PubMed, Scopus, Web of Science, and Google Scholar, were searched for studies published from January 2015 to March 2026. Relevant studies investigating the predefined inclusion criteria related to LOC systems, microfluidic platforms, or biosensor technologies for periodontal or peri‐implant tissues were considered. Two reviewers independently screened records and extracted data from each article on technology type, target biomarkers, sample analyte, reported diagnostic performance, and technology readiness levels (TRLs) by EURAXESS.

**Results:**

Of 1764 records identified, 20 studies met the inclusion criteria. Saliva was the most common sample matrix, followed by gingival crevicular fluid (GCF) or subgingival bacterial templates. The platforms ranged from paper‐based microfluidics and electrochemical or optical biosensors to continuous‐flow PCR chips and most targeted red‐complex periodontal pathogens or host inflammatory markers such as MMP‐8 and IL‐1β. Clinical diagnostic accuracy was rarely reported, validation was usually conducted under controlled laboratory conditions rather than in clinical settings, and most devices remained at the prototype stage.

**Conclusion:**

LOC technologies show clear analytical promise for periodontal diagnostics, but laboratory prototypes still dominate the evidence base. Multicentric clinical validation, standardized fabrication protocols, and clearer regulatory pathways are the main steps required before routine clinical use.

## 1. Introduction

Periodontitis is a chronic inflammatory disease of the gingiva and the tooth‐supporting structures. Its onset reflects a complex interaction between bacterial pathogens, the host immune response, and environmental factors, and, left untreated, the progressive breakdown of the alveolar bone and periodontal ligament can end in tooth loss [1]. The burden is substantial. More than 700 million people worldwide live with severe periodontitis, and because the disease is now linked to systemic conditions such as diabetes mellitus, cardiovascular disease, and adverse pregnancy outcomes, early and accurate detection has become essential to preventing irreversible damage [2].

Conventional diagnosis relies on clinical examinations, together with digital imaging and radiographs. These methods are useful, but they largely record past tissue destruction rather than the inflammatory activity underway, which limits any prediction of future progression. Bleeding on probing (BOP) illustrates the problem: a positive site progresses to attachment loss in only about 30% of cases, whereas a negative result predicts stability in ~98% of the cases [3–7]. A reliable test capable of identifying individuals or sites at risk before clinical tissue damage occurs remains an important unmet clinical need. Saliva and gingival crevicular fluid (GCF) are the plausible biofluids of choice for noninvasive detection of periodontal inflammatory biomarkers [8].

Laboratory assays remain the analytical gold standard, yet developing tests that are simultaneously sensitive, specific, accessible, and affordable enough for routine clinical use remains challenging. Therefore, there has been a growing interest in point‐of‐care testing (POCT), which allows biological samples to be analyzed immediately at the chairside [9]. Lab‐on‐a‐chip (LOC) technology underpins this method by integrating microfluidics and biosensing components into a single miniaturized platform capable of performing multiple laboratory functions [8]. Fundamentally, these microplatforms enable rapid, multiplex detection and quantification of host‐derived inflammatory cytokines, salivary biomarkers, and specific periodontal pathogens, allowing real‐time assessment of periodontal status without the need for central laboratory analysis. This technology supports personalized diagnosis, early detection, and longitudinal monitoring of periodontal disease activity [10–14].

Although research on LOC and microfluidic technologies in oral healthcare is expanding, evidence on their diagnostic applications and translational readiness in periodontology and peri‐implant tissues has not been systematically mapped. This review aims to explore the current evidence on the design, diagnostic performance, and clinical translation of these technologies. It basically examines the LOC and microfluidic platforms developed for periodontal and peri‐implant diagnostics, the biomarkers they target, their stage of clinical translation, and the key knowledge gaps that should guide future research.

## 2. Methods

### 2.1. Study Design

The review followed the Joanna Briggs Institute (JBI) methodology for scoping reviews and is reported against the Preferred Reporting Items for Systematic Reviews and Meta‐Analyses Extension for Scoping Reviews (PRISMA‐ScR) checklist. This design was chosen because the evidence is broad, heterogeneous, and multidisciplinary. The review aimed to map the use of LOC and microfluidic platforms for the diagnosis of periodontal and peri‐implant diseases by characterizing the biomarkers and biological targets investigated and assessing the stage of clinical translation of each technology. Although the review was not prospectively registered, a predefined in‐house protocol was developed before the literature search.

### 2.2. Formulating Research Question

The research question for the current review was as follows: what is the current state of evidence on the operational components, diagnostic capabilities, and translational readiness of LOC and microfluidic technologies in clinical periodontology and implantology?

### 2.3. Search Strategy

The database search was conducted in PubMed (MEDLINE), Scopus, Web of Science, and Google Scholar, with a publication date range from 2015 to March 2026. MeSH terms were combined with free‐text keywords using Boolean operators. The full string applied across databases was: ((“lab‐on‐a‐chip” OR “microfluidic ^∗^” OR “organ‐on‐a‐chip” OR “biosensor ^∗^” OR “aptasensor ^∗^” OR “bioreactor ^∗^” OR “point‐of‐care” OR “microchip”) AND (“periodont ^∗^” OR “gingiv ^∗^” OR “peri‐implant ^∗^” OR “gingival crevicular fluid” OR “peri‐implant crevicular fluid” OR “periodontal ligament” OR “dental implant ^∗^” OR “salivary biomarker ^∗^”)) OR (“tooth‐on‐a‐chip” OR “implant‐on‐a‐chip” OR “implant‐on‐chip”).

### 2.4. Eligibility Criteria

Eligibility was defined a priori using the JBI population–concept–context (PCC) framework.

#### 2.4.1. Population

Eligible studies evaluated or simulated human periodontal and peri‐implant tissues in health and in inflammatory disease, including gingivitis, periodontitis, and peri‐implantitis. Cell cultures, organoids, microfluidic tissue models, relevant animal models, and clinical participants were all considered.

#### 2.4.2. Concept

The concept of interest was the miniaturized total analysis system (µ‐TAS): LOC architectures, microfluidic channels and networks, micropumps, microvalves, microreactors, and integrated optical, electrochemical, or surface‐acoustic‐wave biosensors. Related formats such as microfluidic paper‐based analytical devices (µPADs) and lateral flow assays were also included.

#### 2.4.3. Context

The context comprised chairside POCT, molecular diagnosis of periodontal and peri‐implant disease from oral biofluids (saliva, GCF, and peri‐implant crevicular fluid [PICF]), in vitro models of periodontal disease, and translational work aimed at clinical deployment.

#### 2.4.4. Inclusion and Exclusion Criteria

Studies were eligible for inclusion if they evaluated or applied LOC, microfluidic, biosensor, or other miniaturized analytical technologies in periodontal or peri‐implant tissues, diseases, or clinically relevant experimental models; were published in English; and appeared between January 2015 and March 2026. Studies were excluded if they focused exclusively on nonperiodontal oral diseases without relevance to periodontal or peri‐implant biology, were review articles, systematic reviews, scoping reviews, metaanalyses, editorials, commentaries, opinion papers, letters, or conference abstracts, or were published outside the predefined date range or in languages other than English.

### 2.5. Study Selection

To create a thorough search strategy, a preliminary search on PubMed was performed to review the words used in the titles and abstracts, as well as any index keywords. Following which PubMed, Scopus, Google Scholar and Web of Science were checked using all chosen keywords and index terms. Following the search, all identified citations were collected and uploaded into Rayyan software (http://rayyan.qcri.org) [15], and duplicates were removed. Finally, the reference lists for all articles were checked, and additional relevant studies that met the inclusion criteria were found and incorporated for the review. A meticulous two‐reviewer approach was employed to ensure the inclusion of relevant studies. Abstracts that did not provide enough information for inclusion were excluded. After selection, two researchers (MM and JV) independently screened the titles and abstracts against the PCC criteria. Furthermore, the full text of potentially appropriate articles was assessed. A third reviewer (JJ) resolved disagreements at either stage. The entire study selection process is shown in the PRISMA flow diagram (Figure 1).

**Figure 1:**
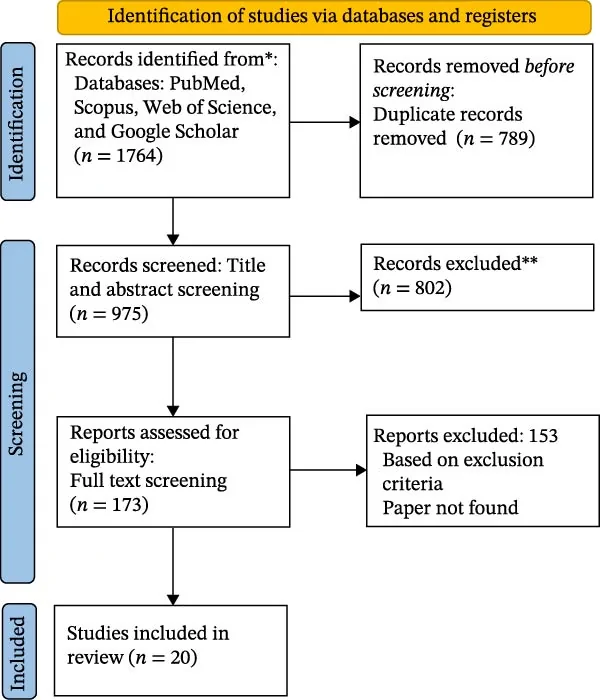
PRISMA 2020 flow diagram for new systematic reviews that included searches of databases and registers only.

### 2.6. Data Charting and Synthesis

Two reviewers (MM and JV) independently extracted data using a standardized table in Microsoft Excel form. The fields recorded were first author and year, study design and setting, technology type and platform, target analyte or biomarker, sample matrix (saliva, GCF, PICF, cell‐culture medium, or other), reported diagnostic performance (sensitivity, specificity, limit of detection, and correlation with a reference standard), disease context, and translational readiness (proof‐of‐concept, prototype validation, or clinical study) (Table 1).

**Table 1:** Standardized data extraction framework utilized for included studies.

Sl. number	Source	Reference	Target analytes and sample	Platform and fabrication materials	Detection principle and time	Analytical performance (limit of detection: LOD, sensitivity, and specificity	Conclusion	Technology readiness levels by EU
1	Baumgartner et al., 2021	[16]	Target: 10 oral bacteria (including *A.a*, *P.g*, *T.d*, and *T.f*) Sample: Whole saliva	OralDisk (centrifugal microfluidic platform) Materials: Polycarbonate polymer foils, magnetic beads, and qPCR master mix	Presence/absence of 10 oral bacteria via DNA Time: <3 h	LOD: ~Cq 24 positive predictive agreement: 88.0% (vs. lab‐based reference) Negative predictive agreement: 88.1% (vs. lab‐based reference)	The OralDisk achieved a ~90% qualitative agreement with standard lab‐based reference methods for detecting both periodontal and cariogenic bacteria. This demonstrates the platform’s potential and suitability for inclusion in larger prospective implementation studies in point‐of‐care dental settings	TRL 6
2	Hassani et al., 2026	[17]	Target: Oral polymorphonuclear neutrophils (oPMNs) Sample: Oral rinse (saline swish)	Cap‐oPMN (capillary microfluidics with YOLOv4 deep learning) Materials: Hycrophilic polyester films, PETE membranes (20 μm)	Automated image processing/counting of oPMNs Time: <30 s (enumeration); ~10 min (total sample‐to‐result)	The platform uses single‐class object detection (looking only for oPMNs against an empty fluid background) The study relies on detection precision—95% detection precision rate 100% isolation purity for single oral neutrophils (oPMNs), achieving near‐perfect concordance with gold‐standard counting (CCC ≈ 0.999)	The Cap‐oPMN platform provides a platform for Rapid quantification without staining and equipment—Free isolation of oral neutrophils and A.I based analysis for real‐time results under 1 mi. It demonstrated a high reliably stratified inflammatory burden, differentiating advanced periodontal disease from healthy and early‐stage conditions	TRL 6
3	Liu et al., 2021	[18]	Target: C‐reactive protein (CRP) Sample: PBS buffer mixed with laboratory antigen and antibody	3D capacitive biosensor with integrated microfluidics Materials: Gold electrode arrays, SU‐8, PDMS, and anti‐CRP antibody	Label‐free real‐time capacitance changes (nF/pF) Time: Not mentioned, but states real‐time detection	LOD: 1 pg/mL Sensitivity: High Specificity: Good	The microfluidic‐integrated 3D capacitive biosensor successfully achieved real‐time, label‐free detection of CRP down to 1 pg/mL. Its stable and effective responses when tested in serum demonstrate its potential for clinical application in cardiovascular and periodontitis biomarker detection	TRL 5
4	Kim et al., 2025	[19]	Target: *S. mutans*, *P. gingivalis*, *C. rectus*, and *P. nigrescens* Sample: Standard gDNA and artificial saliva	Plasmonic photothermal digital PCR (dPCR) chip Materials: OPE‐modified gold nanofilm, glass substrate, and PDMS	Multiplex bacterial gDNA absolute quantification via fluorescence Time: 23 min	LOD: 10^1^ copies/µL (analytical) Sensitivity: High Specificity: High	The ultrafast plasmonic photothermal‐based digital PCR platform is highly sensitive and successfully amplified multiplexed target genes of periodontal pathogens within 23 min. It demonstrates strong potential to be utilized as a point‐of‐care molecular diagnostic device for the ultrafast diagnosis of diseases	TRL 4
5	Yang et al., 2023	[20]	Target: *P. gingivalis*, *T. denticola*, *T. forsythia*, and *E. coli* Sample: Bacterial DNA templates	Multiplex circular array continuous‐flow PCR (CF‐PCR) Materials: PDMS, glass substrate, SU‐8, and SYBR Green I	Fluorescence intensity of amplified PCR products Time: 23 min	LOD: 2.5 × 10^−3^ ng/µL (*E. coli*) Sensitivity: High Specificity: High	An integrated microfluidic chip successfully transported PCR products directly to an electrophoresis microchannel for immediate separation. It achieved amplification and detection of *P.g*, *T.d*, and *T.f* within approximately 11 min, demonstrating significant advantages in speed and convenience for integrated POCT platforms	TRL 4
6	Li et al., 2021	[21]	Target: *P. gingivalis*, *T. denticola*, and *T. forsythia* Sample: Gingival crevicular fluid (GCF) supernatant	CF‐PCR microfluidic chip with integrated heaters and CE Materials: SU‐8, PDMS, glass slide, and TBE buffer	Multi‐PCR gene amplification was followed by capillary electrophoresis Time: 3 min 48 s to 8 min 25 s	LOD: Sensitivity: Not mentioned Specificity: Not mentioned	The double‐layer CF‐PCR microfluidic chip successfully detected bacteria from g,c.f. within 12 min 33 sec, offering a rapid new way for detecting bacteria from G.C.F	TRL 4
7	Yang et al., 2022	[22]	Target: *P. gingivalis*, *T. forsythia*, and *T. denticola* Sample: Gingival crevicular fluid (GCF)	CF‐PCR array microfluidic chip Materials: PDMS, SU‐8, silicon wafer, and aluminum heating blocks	Target gene (DNA) amplification Time: <8 min 5 s	LOD: Amp time: 2 m 7 s (*P.g*), 2 m 51 s (*T.d*), and 5 m 32 s (*T.f*) Sensitivity: Not mentioned Specificity: Not mentioned	A CF‐PCR array microfluidic chip divided into three parts successfully amplified *P.g*, *T.d*, and *T.f* simultaneously in under 8 min and 5 s. This parallel array method effectively bypasses the cross‐reaction issues of multiplex PCR, aiding the development of high‐throughput POCT	TRL 4
8	Melnik et al., 2026	[23]	Target: Total germ load, *P.i*, *A.a*, *P.g*, *T.d*, and *T.f* Sample: Bacterial cell suspension and DNA standards	FoldChip (roll‐to‐roll fabricated EC/CL foil chips) Materials: PET foils and screen‐printed silver electrodes	Ribosomal RNA via electrochemical or chemiluminescent detection Time: 15 min	Bacterial cells: LOD: 3.4 × 10^4^ cells/µL for *E. coli*, and 5.6 × 10^5^ cells/µL for *P. intermedia* DNA standards 0.01 nM (*P.g*. chemiluminescent); range 0.01–25 nM Sensitivity: High Specificity: High	The R2R fabricated FoldChip demonstrated robust reproducibility and stability. It successfully validated that electrochemical detection provides improved sensitivity (lower limits of detection) over chemiluminescent detection for bacterial cells (*E. coli* and *P.i*), indicating its suitability for scalable point‐of‐care diagnostics	TRL 4
9	Nguyen et al., 2024	[24]	Target: Odontogenic ameloblast‐associated protein (ODAM) Sample: Saliva	IoT‐based microfluidic aptasensor POC device Materials: PMMA, pressure‐sensitive adhesive film, and black PLA	Fluorescence intensity via sandwich‐type binding Time: 30 min	LOD: 0.011 nM Sensitivity: High Specificity: 100%	The IoT‐based microfluidic aptasensor system successfully differentiated between low‐risk and high‐risk periodontitis patients with 100% specificity. It presents a more accessible, user‐friendly, and efficient alternative to traditional methods for rapid, sensitive, and portable detection of ODAM	TRL 6
10	Zhang et al., 2023	[25]	Target: MMP‐8 and IL‐1β Sample: Saliva (artificial and clinical)	Dual‐channel microfluidic electrochemical immunosensor Materials: IrOx nanotubes, 2D MXene nanosheets, and SPEs	Electrochemical signal changes (DPV peak current) Time: 45 min	LOD: 0.13 ng/mL (MMP‐8); 0.014 ng/mL (IL‐1β) Sensitivity: High Specificity: High	The dual‐channel immunosensor utilizing IrOx/Ti3C2Tx nanocomposites successfully achieved highly sensitive, simultaneous detection of MMP‐8 and IL‐1β without cross talk. It accurately differentiated the severity of periodontitis in clinical samples, showing great potential for progression monitoring and chairside treatment guidance	TRL 6
11	Yang et al., 2023	[26]	Target: *P. gingivalis*, *T. denticola*, *T. forsythia*, and *E. coli* Sample: Bacterial DNA templates	Multiplex circular array continuous‐flow PCR (CF‐PCR) Materials: PDMS, glass substrate, SU‐8, and SYBR Green I	Fluorescence intensity of amplified PCR products Time: 23 min (5.38 min fastest single channel)	LOD: 2.5 × 10^−3^ ng/µL (*E. coli*) Sensitivity: Not in source Specificity: 100%	A high‐throughput multiplex circular array CF‐PCR system successfully amplified 12 different target genes and performed on‐site fluorescence detection within 23 min. This approach eliminated cross‐reactions and demonstrated great potential for high‐throughput POCT of harmful bacteria in real life	TRL 4
12	Li et al., 2023	[27]	Target: *Porphyromonas gingivalis* Sample: Bacterial strains of *P. gingivalis*	Double‐layer droplet CF‐PCR microfluidic chip Materials: Mineral oil, tetradecane, SU‐8, and PDMS	Fluorescence signal from amplified target genes Time: 11 min and 16 s	LOD: 0.032 nL droplet volume Sensitivity: Not in source Specificity: High	The double‐layer CF‐PCR microfluidic chip successfully reduced fluidic resistance and minimized bubble generation and reagent evaporation by creating nanoliter droplets. It successfully amplified *P. gingivalis* within 11′16″, offering a rapid new way for detecting bacteria	TRL 4
13	Li et al., 2019	[28]	Target: 16S rDNA of periodontal pathogens Sample: GCF and bacterial strains	Integrated CF‐PCR and electrophoresis biochip Materials: Polycarbonate (PC) chip, PC film, and agarose gel	Target nucleic acid amplification tracking Time: 6 min and 14 s	LOD: 125 cfu/µL Sensitivity: High Specificity: High	The all‐in‐one portable device integrating CF‐PCR and electrophoresis successfully achieved automatic sample injection, rapid DNA amplification, and on‐site PCR product detection for periodontal pathogens in under 4 min, showing great potential for point‐of‐care nucleic acid testing	TRL 4
14	Chen et al., 2026	[29]	Target: *Porphyromonas gingivalis* DNA Sample: *P. gingivalis* DNA	Femtosecond laser‐fabricated PCR device Materials: Silica glass substrate, graphene film, and PDMS cover	DNA amplification (404 bp target sequence) Time: Exact time not given, based on 2 min/cycle and total 36 cycles, at least 72 min	LOD: Not available Sensitivity: High Specificity: N/A—Only 1 bacterial DNA was used	The integration of FS‐laser‐fabricated graphene microheaters on a glass substrate achieved rapid and highly precise thermal cycling (82°C/min). This compact microfluidic PCR device demonstrated high precision and efficiency in DNA amplification, indicating strong potential for POCT molecular diagnostics	TRL 4
15	Leão et al., 2025	[30]	Target: Myeloperoxidase (MPO) and peroxidase (POx) Sample: Unstimulated saliva samples	Microfluidic paper‐based analytical device (μPAD) Materials: Whatman chromatography paper, wax, and chromophores	Colorimetric intensity (RGB scale) of enzyme activity Time: 1 to 30 min	LOD: 7.81 ng/mL Sensitivity: Limited Specificity: Limited	The developed µPAD demonstrated applicability in identifying myeloperoxidase levels in saliva, showing its best results with the TMB chromophore. However, it currently has a limited discriminatory capacity to distinguish between healthy patients and those with periodontal disease, requiring further exploration	TRL 4
16	Sousa et al., 2023	[31]	Target: Nitrite, sAA, pH, and Lactate Sample: Artificial saliva and human saliva (unstimulated)	Dual‐mode paper‐based analytical device (dual‐μPAD) Materials: Whatman quantitative filter paper and vegetal paper, 3D polymer resin, graphite powder, and acetone Cost: <US$0.50/device	Electrochemical (lactate) and colorimetric (nitrite, pH, alpha amylase sAA) Time: 1 to 3 min	LOD: Nitrite: 10.7 μmol/L; sAA: 94.6 U/mL; and lactate: 0.06 mmol/L Sensitivity: Medium Specificity: High	The dual‐μPADs fabricated via a simple 3D‐pen “do‐it‐yourself” protocol successfully provided dual‐mode (colorimetric and electrochemical) biomarker readouts. It successfully differentiated periodontitis patients from healthy individuals based on salivary nitrite, salivary alpha amylase (sAA), pH, and lactate levels	TRL 5
17	Sandetskaya et al., 2019	[32]	Target: Tissue inhibitor of metalloproteinases 1 (TIMP‐1) Sample: Spiked sample and gingival crevicular fluid (GCF)	SPARCL on a chip (fluidic 402) Materials: Cycloolefin polymer (COP)	Flash chemiluminescence (luminescence intensity) Time: 35 min	LOD: 49.3 pg/mL Sensitivity: High for liquid reagent but reduced upon reagent lyophilization Specificity: High	The study successfully demonstrated the first integration of a homogeneous SPARCL immunoassay on a lab‐on‐a‐chip for “mix‐and‐read” quantification of TIMP‐1. While it achieved rapid detection without washing steps, the chip design requires further optimization to improve the precision of quantitative measurements	TRL 4
18	Li et al., 2026	[33]	Target: *Porphyromonas gingivalis* and *Treponema denticola* Sample: Purified DNA	Oscillating‐flow microfluidic PCR Materials: PDMS, glass slides, and polyvinylpyrrolidone (PVP)	Nucleic acid amplification (fluorescence intensity/CE) Time: 20 min	LOD: Not determined Sensitivity: Not determined Specificity: Moderate	The oscillating‐flow microfluidic PCR system successfully achieved reliable amplification of *P. gingivalis* and *T. denticola* samples within 20 min by utilizing polymer‐assisted surface passivation. It offers a balanced combination of speed, flexibility, and compactness, advancing toward practical analytical applications	TRL 4
19	Gaertig et al., 2015	[34]	Target: 16S rRNA of 10 periodontal 16S rRNA Sample: DNA isolated from pathogens	Rotating Lab‐on‐a‐chip PCR system Materials: Polycarbonate PCR chip, sealing foils, and lyophilized reagents	Species‐specific DNA sequence fluorescence tracking Time: 40 to 60 min	LOD: 2 to 20,000 copies/reaction Sensitivity: High Specificity: High but readout reliability is compromised by bubble formation	The rotating lab‐on‐a‐chip system enables the fast detection of periodontal pathogens. By adding trehalose to the PCR mix, reagents could be successfully lyophilized and stored at room temperature for months without losing polymerase activity, reducing costs and simplifying diagnostics for dental offices	TRL 4
20	Li et al., 2024	[35]	Target: *Porphyromonas gingivalis* Sample: DNA template of *P. gingivalis*	Microchamber PCR microfluidic chip (rotating platform) Materials: PDMS, glass substrate, aluminum heaters, and nylon Cost: US$60 for a full system build	Target gene amplification (fluorescence intensity) Time: 35 min	LOD: 1.05 × 10^1^ copies/µL Sensitivity: High Specificity: High	The low‐cost and self‐assembled microchamber PCR platform successfully amplified the *P. gingivalis* target gene within 35 min. Its low cost, ease of miniaturization, and light weight give it significant potential for practical bacterial diagnosis in remote areas	TRL 4

Given the heterogeneity in design, technology, and outcomes, data were synthesized narratively in line with the JBI guidance. The included studies were LOC‐based salivary and GCF diagnostics. A narrative summary was used to evaluate the technical and clinical landscape. Technologies were grouped by platform type to compare their reported diagnostic performance (sensitivity, specificity, and limit of detection). Furthermore, the structural maturity and clinical potential of the devices were mapped using the EURAXESS‐extracted technological readiness level, which had a 9‐point scale [36].

## 3. Results

### 3.1. Search Results and Study Selection

The search strategy and study selection process were conducted in accordance with the PRISMA guidelines and are summarized in the PRISMA flow diagram (Figure 1). The database search identified a total of 1764 records, comprising 796 from Scopus, 719 from Web of Science, 195 from PubMed, and 54 from Google Scholar. Following the elimination of duplicates, 975 single records remained for title and abstract screening, of which 802 were excluded for not meeting the predefined eligibility criteria. The full texts of the remaining 173 articles were assessed for eligibility based on the PCC framework. Of these, 153 studies were excluded, primarily because they did not evaluate or apply LOC or microfluidic technologies within a periodontal or peri‐implant context or because they were review articles, editorials, conference reports, or other publications lacking original research data. Consequently, 20 studies met the eligibility criteria and were included in the final comprehensive review.

### 3.2. Descriptive Characteristics of Included Studies

The included literature clusters around two goals: miniaturizing nucleic acid amplification and monitoring the host immune response in periodontal and peri‐implant settings. Human or simulated saliva was the most common matrix, followed by GCF and/or subgingival bacterial templates, with the remainder using purified DNA templates or cell suspensions.

Among the significant studies, analytical performance characteristics, particularly limit of detection and amplification time, were reported substantially more frequently than measures of clinical diagnostic accuracy, highlighting a gap in clinically relevant validation. Sensitivity and specificity against an accepted reference standard were absent from most records (Table 1). For example, performance metrics such as 100% specificity predominantly exhibit analytical validation of the assay rather than its clinical diagnostic performance. Even the apparent exception of 88.0% positive predictive agreement and 88.1% negative predictive agreement reported for OralDisk signifies agreement with the lab‐based reference assay and should therefore be interpreted as an analytical consensus rather than diagnostic precision against an established clinical disease reference standard [16].

### 3.3. Thematic Synthesis of Domains

#### 3.3.1. Chairside and Paper‐Based Diagnostic Platforms

Paper‐based microfluidic devices (µPADs) occupy the most accessible form of POCT, utilizing passive capillary‐driven fluid transport to achieve affordable diagnostics with minimal instrumentation. Leão et al. [30] built a low‐cost µPAD from chromatography paper and wax barriers that quantifies myeloperoxidase (MPO) and peroxidase (POx) colorimetrically straight from unstimulated saliva, reaching a detection limit of 7.81 ng/mL within a one‐to‐thirty‐minute window. Sousa et al. [31] took a “do‐it‐yourself” route, using filter paper, vegetal paper, and 3D‐pen resin to lay out the fluidic paths; their dual‐mode device paired electrochemical lactate readout with colorimetric measurement of nitrite, pH, and salivary alpha‐amylase (sAA) in under 3 min. These platforms show the requirement of limited hardware and chairside screening. Their reliance on hand fabrication and ambient‐light‐sensitive colorimetry, however, already foreshadows the reproducibility limits discussed below.

#### 3.3.2. LOC‐Based Salivary and GCF Diagnostics

Saliva‐ and GCF‐based LOC platforms primarily employ immunochemical or biochemical assays to quantify host inflammatory biomarkers. Zhang et al. [25] developed a dual‐channel electrochemical immunosensor using screen‐printed electrodes (SPEs) functionalized with iridium oxide nanotubes (IrOx NTs) and MXene nanosheets, enabling simultaneous detection of matrix metalloproteinase‐8 (MMP‐8) and interleukin‐1β (IL‐1β) within 45 min, with detection limits of 0.13 ng/mL and 0.014 ng/mL, respectively. For GCF analysis, Sandetskaya et al. [32] used an on‐chip SPARCL immunoassay to detect tissue inhibitor of metalloproteinases 1 (TIMP‐1) via flash chemiluminescence, achieving a detection limit of 49.3 pg/mL in 35 min. Although these platforms directly assess host inflammatory responses and may better reflect disease activity than pathogen‐based assays, their application remains limited, and validation has largely been confined to artificial matrices or small clinical cohorts.

#### 3.3.3. Microfluidic Systems for Pathogen Amplification

Microfluidic molecular diagnostic platforms integrate nucleic acid extraction, amplification, and detection within a single miniaturized device. Rapid DNA amplification is achieved by flowing the PCR reaction mixture through microchannels maintained at different temperature zones, markedly reducing the assay time compared with conventional PCR. Chip‐based molecular diagnostics concentrate on the destructive red‐complex periodonto‐pathogens that is *Porphyromonas gingivalis*, *Treponema denticola*, and *Tannerella forsythia*. Two studies by Li et al. [21, 27] and Yang et al. [22] demonstrated the application of continuous‐flow polymerase chain reaction (CF‐PCR) microchips to substantially reduce conventional thermal cycling times. These platforms achieved rapid nucleic acid amplification by continuously routing samples through microfluidic channels across patterned aluminum microheaters or integrated microchip capillary electrophoresis (MCE) systems, enabling multiplex detection of periodontal pathogens within 3–12 min with detection limits of ~10–100 bacterial cell copies. Chen et al. [29] further improved thermal performance by incorporating femtosecond laser‐fabricated graphene microheaters into the microfluidic PCR platform, while Kim et al. [19] developed an ultrafast plasmonic photothermal digital PCR (dPCR) chip capable of multiplex pathogen detection within 23 min. Collectively, PCR‐based microfluidic platforms represent the most technically advanced and extensively investigated category identified in this review. However, these systems predominantly focused on qualitative identification of periodontal pathogens rather than quantitative assessment of disease activity or host inflammatory status. Furthermore, relatively few studies validated their diagnostic performance against conventional quantitative PCR (qPCR), limiting direct assessment of their clinical accuracy and translational applicability [16, 19].

#### 3.3.4. Digital Integration Technologies

Coupling automated analysis with microfluidics is an emerging direction. Hassani et al. [17] designed the Cap‐oPMN platform, a self‐powered capillary chip that isolates oral polymorphonuclear neutrophils (oPMNs) from a saline rinse and counts them with a YOLOv4 deep‐learning detector at single‐cell resolution. Nguyen et al. [24] addressed connectivity instead, building an internet‐of‐things (IoT) microfluidic aptasensor for odontogenic ameloblast‐associated protein (ODAM) in saliva that reached a 0.011 nM detection limit in 30 min through a dedicated optical module. Both point toward automated, networked chairside testing, though each rest on a single proof‐of‐concept report.

### 3.4. Translational Readiness and Clinical Maturity

The studies were evaluated using the EURAXESS technology readiness level (TRL) scale to assess the current state of clinical translation, which revealed a pronounced concentration of devices in the early to midstage of development. Among the 20 included studies, the majority were classified at TRL 4, indicating that the systems integrate basic technological components that work together at low fidelity in controlled laboratory environments using purified targets, D.N.A. primers, purchased biochemicals, etc. Two studies were positioned at TRL 5, which represented the integration of components with reasonable and realistic fidelity while testing in a simulated environment or using real samples for initial validation. Four studies advanced to TRL 6, demonstrating successful prototype demonstration in a relevant clinical setting or with clinical samples (e.g., whole saliva, oral rinse, GCF, and plaque samples). No platforms in the current dataset reached TRL 7 or above, which is a critical gap between proof‐of‐concept analytical engineering and regulatory or commercial clinical readiness in periodontology. The graphical representation of the TRL distribution percentage of the studies included is present in Figure 2.

**Figure 2:**
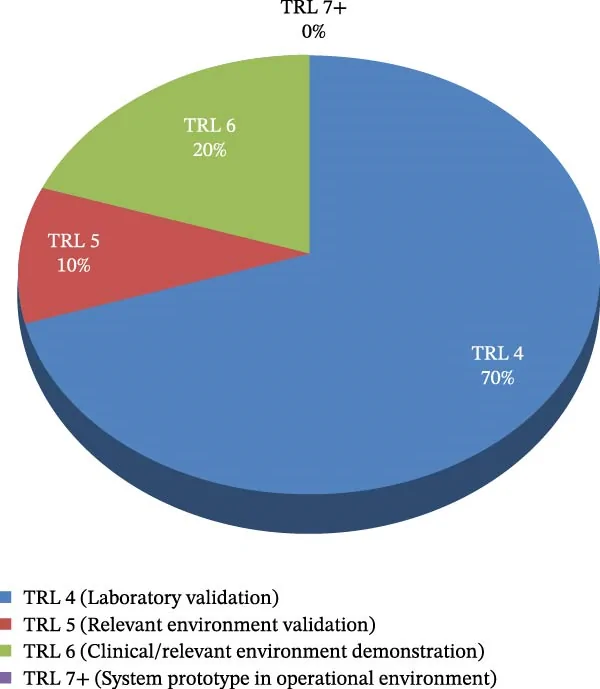
Distribution of the included studies according to their translational readiness, classified using the EURAXESS technology readiness level (TRL) framework.

## 4. Discussion

### 4.1. Summary of Principal Findings

This review mapped the global evidence on microfluidic and LOC technologies in periodontology. Two engineering priorities dominate: miniaturizing nucleic acid amplification (CF‐PCR and dPCR) and building sensitive biochemical sensors for classical pathogens and host cytokines such as MMP‐8 and IL‐1β, mostly from saliva and GCF. Set against that technical purpose, however, is a clear gap between what these devices can detect and what they have been shown to do in routine clinical care. Beyond the technical performance of these technologies, the strength of the supporting evidence and the paucity of cost‐effectiveness data remain major determinants of their translational readiness for clinical practice. These issues are examined in the following sections.

### 4.2. Translational Readiness and Clinical Applicability

This review distinguishes three concepts that are often used interchangeably but present different stages of technology development: commercially available point‐of‐care tests, clinically validated prototypes, and experimental laboratory proof‐of‐concept platforms. Most of the 20 included studies belong to the proof‐of‐concept stage. Although many reported effective analytical performances, including detection of biomarkers at picogram concentrations and highly sensitive pathogen detection, these findings were generally obtained under controlled laboratory conditions using purified DNA, artificial saliva, or small, selected clinical samples. Therefore, these results should not be interpreted as evidence of clinical readiness since none of these integrated platforms have reached routine periodontal practice or established within standard clinical workflows.

### 4.3. Critical Appraisal of the Evidence Base

Consistent with JBI scoping‐review methodology, no formal risk‐of‐bias tool was applied to individual studies; what follows instead is a narrative appraisal of recurring features of study design that informs on how the evidence should be interpreted. Beyond the translational stage, the certainty that can be placed in the evidence itself is limited, for reasons that are consistent across the dataset rather than specific to any one platform.

• Study design: All included studies were single‐arm technical or analytical validation studies. None of the studies are structured as a diagnostic accuracy study with a prespecified index test, a blinded comparison against a reference standard, or a calculated sample size, which is the design needed to support a clinical performance claim.
• Sample resource: A substantial share of the platforms in Table 1 were validated on purified DNA templates, bacterial cultures, or artificial saliva rather than patient‐derived material; where clinical samples were used, cohorts were typically small and drawn from a single center, which limits how far any performance estimate can be expected to generalize.
• Independent replication: Each device was evaluated exclusively by the original laboratory. None of the included studies reported independent validation or direct comparative assessment of previously published data, thus limiting the reproducibility and generalizability across operators, reagent batches, and testing environments.
• Outcome reporting: As Table 1 shows, clinical sensitivity and specificity against an accepted reference standard are marked “not in source” for most studies, while analytical figures such as limit of detection are reported almost universally. This asymmetry suggests that the outcomes that are easiest to demonstrate favorably are also the ones most consistently reported. Hence, there may be a possibility of selective outcome reporting rather than a balanced account of performance.

Overall, the findings suggest that the analytical performance of these platforms appears reliable, but evidence supporting their clinical diagnostic performance remains limited due to small, single‐center studies without adequate validation against established reference standards.

### 4.4. Economic Feasibility

Economic feasibility remains the least discussed aspect of the available literature, despite being a critical feature of whether LOC and microfluidic platforms can be translated into routine periodontal practice. The fabrication materials summarized in Table 1 include a broad and largely qualitative cost spectrum. Most continuous‐flow PCR and dPCR platforms employ cleanroom‐dependent materials such as PDMS, SU‐8 photoresist, and silicon wafers, necessitating multistep photolithographic fabrication and access to specialized microfabrication facilities. Such manufacturing requirements may not be readily available and likely to increase production costs and may limit scalability, particularly in resource‐constrained settings.

Only two studies reported quantitative cost estimates. Sousa et al. [31] estimated a material cost of less than US$0.50 per disposable dual‐µPAD, representing the only reported per‐test consumable cost in the dataset. In contrast, Li et al. [35] reported that their rotating microchamber PCR platform, including the heating system, slide rail, and control software, could be assembled for approximately US$60, representing the cost of the reusable instrumentation rather than the consumable cost per assay. Leão et al. [30] described a similar paper‐based platform as “low‐cost” without providing numerical estimates, while Melnik et al. [23] developed a Foldchip; roll‐to‐roll foil processing designed for high‐throughput, low‐cost production of foil‐based biosensors but likewise did not report unit manufacturing costs.

The remaining studies provided no quantitative information on fabrication costs, materials and per‐test expenses, or economic comparisons with conventional laboratory diagnostics such as qPCR, ELISA, or microbial culture. Moreover, the limited cost data available is not directly comparable. Sousa et al. [31] estimate reflects only the disposable paper substrate and excludes the supporting bipotentiostat and smartphone‐based readout system, whereas Li et al. [35] estimate pertains solely to the reusable hardware platform and does not include consumables required for individual assays. Consequently, neither study provides a comprehensive estimate of the actual cost of chairside testing on a per‐patient basis.

At present, analytical performance remains the primary metric by which these technologies are evaluated. However, cost‐effectiveness is likely to be equally important in determining clinical implementation, particularly in low‐ and middle‐income settings where affordable point‐of‐care diagnostics have the greatest impact. Standardized reporting of manufacturing, instrumentation, and per‐test costs, together with cost‐effectiveness analyses, should become a routine component of future validation studies. Until such data are available, the economic benefits of LOC and microfluidic technologies over conventional periodontal diagnostics cannot be established.

### 4.5. Methodological Limitations of Current Evidence

Several methodological limitations currently block the move from the bench to the chairside.

• Lack of multicentre clinical trials: Most studies rely on small, unstratified samples. Without large, statistically powered validation across diverse cohorts, the real‐world diagnostic accuracy of these platforms—sensitivity, specificity, and predictive values—stays unverified.
• Heterogeneity in reference standards: The lack of standardization in sample collection protocols (for example, stimulated versus unstimulated saliva) along with heterogeneity in periodontal case definitions compromises cross‐study comparability and limits the generalizability of clinical outcomes.
• Absence of longitudinal data validation: Data on long‐term clinical utility and on the shelf life and stability of on‐chip reagents under real storage conditions are almost entirely missing.

### 4.6. Barriers to Implementation and Regulatory Challenges

The implementation of LOC technology in clinical practice, particularly in periodontology and dental implantology, is influenced by several technical, regulatory, economic, and clinical barriers. Although LOC platforms have demonstrated promising diagnostic performance in research settings, their translation into routine clinical use remains limited.

#### 4.6.1. Lack of Standardized Regulatory Approach

Regulatory agencies require extensive evidence of analytical validity, clinical validity, and clinical utility before approving LOC devices. Since many LOC systems integrate microfluidics, biosensors, and artificial intelligence, they often fall under multiple regulatory categories, complicating approval. Regulatory requirements differ across regions (e.g., FDA in the United States and MDR in the European Union), making global commercialization challenging. Additionally, these LOC systems should be reproducible and maintain compliance with quality standards.

#### 4.6.2. Technical Barriers

LOC devices may experience issues like sensor reliability, microchannel clogging, multiplexing limitations, and sample preprocessing, which could reduce their diagnostic reliability. Although multiplexing capabilities are advancing, many LOC devices remain limited to the detection of one or a few biomarkers, with restricted analytical throughput and a need for further optimization for the simultaneous analysis of multiple periodontal biomarkers. Furthermore, contamination of saliva or GCF with cellular debris, blood, or other impurities can impair the microfluidic performance, while the inherent instability of certain biomarkers may compromise diagnostic accuracy and reproducibility.

#### 4.6.3. Clinical Implementation Barrier

The clinical implementation of LOC devices is hindered by several key challenges, including the lack of standardized biomarker panels, limited clinical validation, difficulties in integrating these technologies into routine clinical workflows, and the need for adequate clinician training to ensure their effective use and interpretation.

#### 4.6.4. Digital Challenges

Data security and privacy, software validation, and interoperability with electronic health record (EHR) systems, all of which are essential for the safe and effective integration of LOC technologies into clinical practice.

#### 4.6.5. Translation From Lab to Clinical Practice

The translation of LOC devices from laboratory prototypes to commercial products remains challenging due to variability in saliva and GCF samples, environmental influences, operator‐dependent variability, and limited durability under routine clinical conditions.

### 4.7. Future Directions

Future research should put methodological demands ahead of technical novelty. Studies need to align participant selection and diagnostic indexing with standardized indexed classifications so that results can be compared and, most importantly, to report clinical sensitivity and specificity against an accepted reference standard rather than analytical figures alone. The decisive step will be head‐to‐head clinical trials that benchmark new microplatforms against the current diagnostic practice. Cost‐effectiveness should also become a standard reported outcome alongside analytical and clinical performance since a technically superior device with an unreported or prohibitive per‐test cost offers little practical advantage over the laboratory methods already in use.

### 4.8. Limitations of This Review

The review has its own limits. Restricting the search to English‐language work from 2015 onward may have excluded relevant older or non‐English studies. Gray literature was captured only through Google Scholar, so conference proceedings, patents, and regulatory filings, where early or industry‐led work often sits, may be underrepresented. In line with the JBI scoping‐review method, no formal quality or risk‐of‐bias appraisal was performed, so individual study rigor was not weighted in the synthesis. Finally, because the review was not prospectively registered, some posthoc refinement of the eligibility criteria cannot be ruled out.

## 5. Conclusion

LOC and microfluidic technologies hold real promise for periodontal diagnostics, offering rapid, multianalyte profiling of oral biofluids. For now, though, the evidence is dominated by sensitive but laboratory‐bound prototypes. Closing the distance between engineering innovation and everyday oral healthcare will depend on standardized fabrication, workable regulatory routes, and robust multicenter clinical validation.

**Nomenclature**
*A.a*: *Aggregatibacter actinomycetemcomitans*
A.I: Artificial intelligence
bp: Base pair
cfu: Colony‐forming unit(s)
*C. rectus*: *Campylobacter rectus*
CCC: Concordance correlation coefficient
CE: Capillary electrophoresis
CF‐PCR: Continuous flow polymerase chain reaction
CL: Chemiluminescent
COP: Cycloolefin polymer
Cq: Quantification cycle
CRP: C‐reactive protein
DNA: Deoxyribonucleic acid
dPCR: Digital polymerase chain reaction
DPV: Differential pulse voltammetry
*E. coli*: *Escherichia coli*
EC: Electrochemical
FS‐laser: Femtosecond laser
GCF: Gingival crevicular fluid
gDNA: Genomic deoxyribonucleic acid
IL‐1β: Interleukin‐1 beta
IoT: Internet‐of‐things
IrOx: Iridium oxide
LOD: Limit of detection
MMP‐8: Matrix metalloproteinase‐8
MPO: Myeloperoxidase
MXene: Two‐dimensional transition metal carbide/nitride
ODAM: Odontogenic ameloblast‐associated protein
OPE: Oligo(phenylene‐ethynylene)
oPMNs: Oral polymorphonuclear neutrophils
*P.g*/*P. gingivalis*: *Porphyromonas gingivalis*
*P.i*/*P. intermedia*: *Prevotella intermedia*
*P. nigrescens*: *Prevotella nigrescens*
PBS: Phosphate‐buffered saline
PC: Polycarbonate
PCR: Polymerase chain reaction
PDMS: Polydimethylsiloxane
PET/PETE: Polyethylene terephthalate
PLA: Polylactic acid
PMMA: Polymethyl methacrylate
POC: Point‐of‐care
POCT: Point‐of‐care testing
POx: Peroxidase
PVP: Polyvinylpyrrolidone
qPCR: Quantitative polymerase chain reaction
R2R: Roll‐to‐roll
rDNA: Ribosomal deoxyribonucleic acid
RGB: Red–green–blue
RNA: Ribonucleic acid
rRNA: Ribosomal ribonucleic acid
*S. mutans*: *Streptococcus mutans*
sAA: Salivary alpha‐amylase
SPARCL: Spatial proximity analyte reagent capture luminescence
SPEs: Screen‐printed electrodes
*T.d*/*T. denticola*: *Treponema denticola*
*T.f*/*T. forsythia*: *Tannerella forsythia*
TBE: Tris/Borate/EDTA buffer
TIMP‐1: Tissue inhibitor of metalloproteinases 1
TMB: 3,3′,5,5′‐Tetramethylbenzidine
TRL: Technology readiness levels
µPAD: Microfluidic paper‐based analytical device
YOLOv4: You only look once version 4.

## Author Contributions

**Midhu Manoj**: conceptualization, data curation, writing‐original draft, writing – review and editing; **Jothi Varghese**: project supervision, conceptualization, formal analysis, writing – editing and reviewing; **Josh Jaison**: formal analysis, writing – editing and reviewing.

## Funding

This review did not receive any funding.

## Disclosure

All authors have contributed critically to the manuscript and approved the final version. All authors have read and approved the final version of the manuscript. Jothi Varghese (corresponding author) had full access to all the data in this study and takes complete responsibility for the integrity of the data and the accuracy of the data analysis. All scientific content, data extraction, interpretation of results, and conclusions were developed and verified by the authors, who take full responsibility for the integrity and accuracy of the work.

## Ethics Statement

This article is a scoping review based on published literature and did not involve human participants, patient data, or any intervention. Hence, no ethical clearance was required.

## Conflicts of Interest

The authors declare no conflicts of interest.

## Data Availability

The authors confirm that the data supporting the findings of this study are available within the article and/or its Supporting Information.
